# Evaluating the effect of an educational intervention on the adherence rate to sleep study: A multi-centered stratified randomized controlled trial

**DOI:** 10.1371/journal.pone.0244496

**Published:** 2021-01-07

**Authors:** Shokoufeh Aalaei, Mahnaz Amini, Fariborz Rezaeitalab, Hadi Asadpour, Hamed Tabesh, Farnaz Khoshrounejad, Saeid Eslami, Lahya Afshari Saleh

**Affiliations:** 1 Department of Medical Informatics, Faculty of Medicine, Mashhad University of Medical Sciences, Mashhad, Iran; 2 Lung Diseases Research Center, Faculty of Medicine, Mashhad University of Medical Sciences, Mashhad, Iran; 3 Department of Neurology, Faculty of Medicine, Mashhad University of Medical Sciences, Mashhad, Iran; 4 Sleep Laboratory of Ibn-e-Sina Hospital, Mashhad University of Medical Sciences, Mashhad, Iran; 5 Department of Medical Informatics, University of Amsterdam, Amsterdam, The Netherlands; 6 Pharmaceutical Research Center, School of Pharmacy, Mashhad University of Medical Sciences, Mashhad, Iran; 7 Department of Occupational Medicine, Faculty of Medicine, Mashhad University of Medical Sciences, Mashhad, Iran; Charite Medical University Berlin, GERMANY

## Abstract

An appropriate diagnosis and effective treatment of sleep apnea can improve the associated quality of care and reduce morbidities. The study aims to develop and evaluate an educational intervention tailored to patients’ needs in order to increase the rate of patients’ adherence to physician's prescription for a sleep test. A multi-center, stratified, 2 parallel-arm, randomized controlled trial was conducted. The patients in the intervention group received the educational booklets on sleep apnea and sleep test which was designed based on the extracted factors through an in-depth interview with patients. All participants were contacted after two months to ask whether they completed an assessment for OSA. A total number of 1,650 individuals were screened. Finally, 104 participants were randomized to the control group (n = 50) or intervention group (n = 45) that did not differ significantly in baseline characteristics. The results of the intention to treat analysis indicate that patients in the intervention group were significantly more adherent to attend a sleep assessment for their OSA risk (30%; n = 15/50) than the patients in the control group (11.1%; n = 5/45, P <0.05). Age, history of diabetes, and the educational intervention were effective in performing the sleep test. Time limitations, Condition improvement, and high cost of diagnostic test were the most barriers, respectively. The intervention was successful in improving the adherence rate of patients to prescribed sleep test. However, the adherence rate to sleep study testing is still far from desirable and requires more complex interventions.

## 1. Introduction

Obstructive sleep apnea (OSA) is among the most prevalent sleep disorder marked by temporary obstruction of the upper airway during sleep. This disease adversely affects sleep quality among those afflicted. It also disrupts arterial oxygen and can lead to unfavorable consequences such as making more errors during daily activities, fatal accidents, metabolic syndrome, cardiovascular diseases and even mortalities caused by heart attack or cerebral stroke.

According to the study results by Benjafield and et al. in 2019, the estimations showed that a total number of 936 million adults were between 30 and 69 years old, who showed a mild-severe obstructive sleep apnea (AHI ≥ 5 events/hour). Moreover, 425 million adults were between 30 and 69 years old and had moderate-severe obstructive sleep apnea (AHI ≥ 15 events/hour) on a global scale [1]. In a review study conducted in 2013 among the Asian population, using a questionnaire, the prevalence of sleep apnea was estimated to range between 4.98 and 27.3%. However, polysomnography results showed this rate to range between 3.97 and 7.3% [2]. In the existing literature in Iran, the prevalence of the disorder in different areas has been reported to a range between 5 and 39% [3–5]. As not all patients afflicted with OSA show symptoms of the disease, the number of non-symptomatic patients is larger. Moreover, the prevalence of OSA has grown in recent years due to the rising prevalence of obesity, a key risk factor of OSA.

Correct diagnosis and attempts to treat OSA effectively can help to improve the management of chronic diseases and increase efficiency. A doctor would use the score a patient has acquired from such questionnaires as Epworth Sleepiness Scale (ESS) (to evaluate the degree of sleepiness) [6] and STOP-BANG (to evaluate the risk of OSA) [7] along with physical examinations, medical and family history to decide whether there is a need for the polysomnography (PSG) or not [8]. Polysomnography is the main test for determining the state of night sleep and what occurs during sleep. Observation of the state of sleep and its features can reveal many sleep disorders and factors affecting sleep and can, thus, make treatment possible.

Despite the high prevalence and consequences of apnea, there are some patients do not follow their doctors’ advice to undergo the sleep test. In previous studies, the rate of non-adherence to doctors’ advice to undergo PSG has been reported to range between 7–81.7% [9–12].

Different factors such as the high cost of test, unavailability of services, lack of family support, unfamiliar environment, being observed during sleep, work obsessions, traffic problems, affliction with other diseases, stress concerning polysomnography test, and lack of support by the clinical team are mentioned as the barriers to the conduction of the sleep test [12–15]. Different studies have explored the effect of interventions on increasing the adherence rate to physician order on conducting the sleep test [11, 15–17].

Considering the expansive spread of apnea, there is a need for purposeful interventions to increase the adherence rate to prescribed sleep test. Thus, it is essential to explore the target patients’ concerns, attitudes, and perspectives about sleep test administration before developing any intervention.

Therefore, the present research aims to develop and evaluate an educational intervention tailored to patients’ needs in order to increase the rate of patients’ adherence to physician's prescription for a sleep test.

## 2. Method

### 2.1. Study design and participants

A multi-center, stratified, two parallel-arm, randomized controlled trial was developed to compare the enhanced rate of patients’ (suspects of sleep apnea) adherence to physician's prescription for sleep test using tailored educational booklets with usual care. This study was approved by the Ethics Committee of Mashhad University of Medical Sciences (Ethical code: IR.MUMS.fm.REC.1395.571). Also, it was registered at IRCT.ir (IRCT20170922036314N3). All participants provided oral and written informed consent.

**Fig 1:**
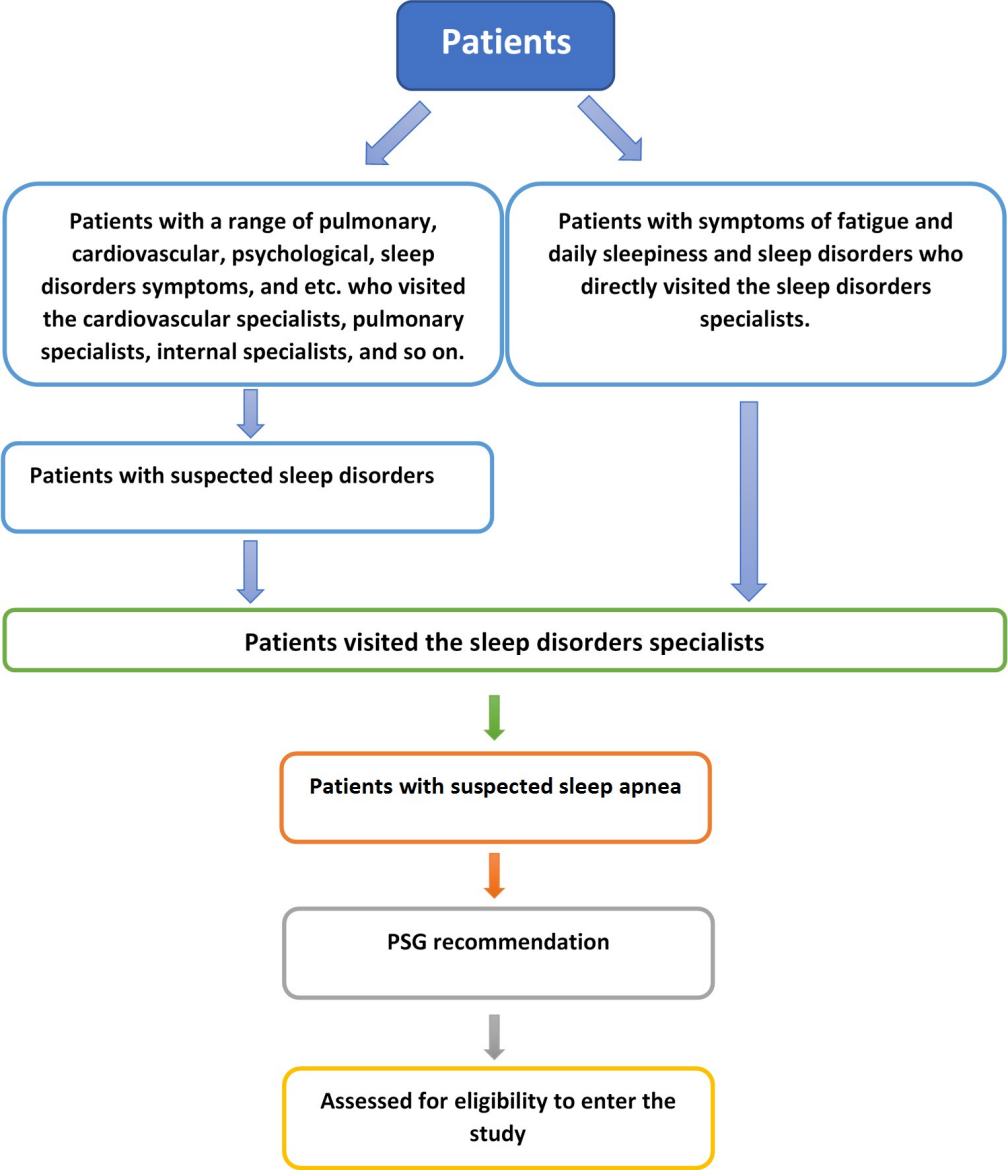
The initial process of patients' selection for the study.

Between January 2018 and August 2018, all adult patients referred to three university clinics (including a general clinic, a sleep center in the hospital, and an occupational medicine center) and a private office in Mashhad, Iran's second metropolis. The patients were visited by a sleep medicine specialist in each center and were screened for eligibility. Fig 1 shows the initial process of patients' selection for the study. Geographically, the centers were located in different parts of the city and, thus, patients’ socioeconomic status varied. As for the professional experience, no significant difference was found between the visiting doctors. As for gender, two doctors were male and two were female.

### 2.2. Eligibility criteria

Eligible patients were required to be at or above 18 years of age, accessible by telephone, with baseline literacy, suspected with sleep apnea according to the AASM guideline and physician examination and having referred for a sleep test (split/full-night PSG) by a physician, not forced to perform sleep test for employment purposes, without a previous diagnosis of dementia or sleep apnea, not at risk of other sleep disorders (e.g., severe insomnia), with no prior PSG or HST. Patients who were unable or unwilling to provide informed consent or not accessible at the end of the study were excluded.

### 2.3. Randomization and enrollment

Because of the inherent divergence of centers and physicians' behavior in treating patients, center-stratified randomization was followed to assign patients to control and intervention arms (allocation ratio: 1:1). Therefore, the patients were stratified by the clinical center. This strategy would ensure a balance between the two study arms regarding patients being included from different centers. Without such stratification, samples could be biased towards a crowded center. Thus, there were four strata with equal control and intervention samples.

The randomization sequence was created using www.randomization.com. The generated random numbers were transferred to sealed envelopes by an independent researcher, not involved in the data collection or intervention procedure. Each set of envelopes was given to a predetermined coordinator in each center. Corresponding envelopes were opened only after the target participants signed the informed consent, completed all baseline assessments, and the right time to form the intervention group.

### 2.4. Standard care

In each center, eligible patients received standard care including physical examination, comprehensive evaluation of sleep disorders especially OSA, education on sleep test and treatment procedures during a visit by the physician. After the visit, eligible patients were told about the purpose of the research and were ensured by the physician that they were to be included in the study upon their consent.

### 2.5. Baseline measurements

If patients showed interest to participate in the study, baseline data including socio-demographics, anthropometric data (i.e., height and weight), medical history, diseases associated with risk for OSA (hypertension, diabetes, heart disease, stroke, etc.), family history of sleep problems, history of a car accident, the Iranian version of Epworth Sleepiness Scale (ESS-IR) score [18], the STOP-BANG score were added to a form by the physician.

ESS is used to evaluate the state of sleepiness for individuals that can range between 0 and 24 based on patient response. STOP-BANG is used to evaluate the rate of affliction with OSA that can range between 0 and 8.

After completion of all baseline assessments for the enrolled participants, informed consents were obtained. Then the coordinator opened an envelope and accordingly assigned a patient to the control or intervention group. Patients in the latter were provided with tailored educational materials including "Sleep Apnea" and "Sleep Test" booklets.

Contact information was taken from each patient for the follow-up.

### 2.6. Interventions

To develop and implement the educational material in accordance with the needs and expectations of the target population, three phases including need analysis, design and implementation and evaluating content quality were conducted.

#### 2.6.1. Phase 1: Need analysis

Initially, field observation was used to gain an overall view of patients’ information needs. To this aim, one of the present researchers (first author) attended, for a month, the sleep specialist-patient visits in two university centers. Then, some qualitative research, content analysis in type, was conducted to extract the influential factors in sleep test administration by patients.

In this phase, in-depth semi-structured interviews were conducted. Individual face-to-face interviews were conducted. For the sampling, two university clinics were selected in Mashhad, Iran, to which patients were visited by the sleep medicine specialists. All the attending patients were suspected of sleep apnea according to the AASM guideline and were advised to undergo a night sleep study. At the outset, they were selected through a purposeful sampling method. The sampling continued up until data saturation occurred. All interviews took place at a room beside the visiting room in the clinics, which was equipped with a computer system and the necessary equipment to conduct the interviews.

At the beginning of each interview, the purpose of the research was revealed to all participants and they were ensured that their participation was upon their consent. They were also told in advance that their voice would be recorded during the interview and all information they provided during the interview was to be kept confidential and served only for research purposes. Their informed consent was subsequently obtained.

To ensure full coverage of the topic of research and an organized interview, the initial plan was designed by the present researchers. At the end of the interviews, each patient was asked whether s/he was willing to take the sleep test. If the answer was negative, then the main question was asked to explore the inhibiting factors, "What factors dissuade you from taking the sleep test?" During the interviews, in-depth questions or exploratory items were asked. It is noteworthy that the patients' concepts were prioritized to the pre-planned items. Therefore, patients’ descriptions were not interrupted at all. When needed, the order of questions changed in accordance with the patients’ commentaries. Occasionally, new questions were raised to extract more detailed information out of the patients' statements.

As soon as the interview was over, the acquired data were transcribed in Microsoft Word. For data coding, categorization and analysis, the interview files entered MAXQDA 10. The content of the interview was then coded according to the concepts corresponding to the purpose of the research. It should be noted that the consensual opinions of the multi-disciplinary research team members were used in all processes of data collection and analysis.

Overall, 12 participants were interviewed. Analysis of data revealed that different factors influenced patients' decision to adhere to the doctors’ prescribed advice. Four main categories of barriers were identified including: inadequate knowledge, psychological factors, cost and the service system. Each category and its respective subcategories are shown in Table 1.

**Table 1:** Extracted categories and subcategories from the in-depth semi-structured interviews.

Category	Subcategory
Inadequate knowledge	Inadequate knowledge of the disease symptoms
	Not accepting the disease
	Not prioritizing the sleep test over other medical fees
	Perceiving the symptoms as natural phenomena
	Unawareness of the post-test treatment
	Not considering the disease and its consequences seriously
	Time limitations due to job-related commitments
	Worries of monitored dreams
Psychological factors	Stressful devices
	Fear of the test device and environment
	User-unfriendliness of the device
	Discomfort with an unfamiliar environment.
	Reluctance to attend hospital centers
	Personal traits
Cost	High cost of the diagnostic and therapeutic test
	Lack of insurance coverage (social support)
	No family support to pay the costs
Service system	Geographic distance to the facility
	Time-consuming process of making appointment and conducting the test
	Dissatisfaction with the hospital services and personnel

As the results point out, the factors affecting adherence to the physician's prescription are divided into two categories. In the first category, cost and service system factors should be addressed at higher levels by policymakers. Efforts to reduce costs through insurance coverage or subsidization and promote quality of service provided to the patients can play an essential role in patients’ willingness to undergo the test.

On the other hand, knowledge and psychological-related factors are modifiable factors that can be intervened at the patient level [19]. In this way, we believe implementing educational interventions to enhance patients’ awareness and address their concerns can tremendously increase the rate of patient’s adherence.

After the results were discussed by experts in the light of their prior experience of patients’ needs, the educational content was decided to be presented in two parts: disease and diagnostic test.

#### 2.6.2. Phase 2: Design and implementation

To meet users’ needs, it was decided to present the educational content as a mixture of text and image in two booklets named as “Sleep Apnea” and “Sleep Study”.

In the “Sleep Apnea” booklet, it was attempted to highlight the problem and probable adverse effects of the disease on patients and increase their knowledge, risk perception, and motivation to participate in the diagnosis and treatment procedures. Thus, the definition, symptoms, risk factors, and adverse effects of sleep apnea were considered as the core content of this booklet. In “Sleep Study”, the psychological barriers to sleep test administration were focused. It was attempted to elaborate on the reason for test administration, the environment, the manner of administering the test, and how to analyze the test results to remove all patient’s ambiguities with this concern.

To provide the educational content, credible global websites, published materials, and specialists’ comments were used.

#### 2.6.3. Phase 3: Evaluating content quality

To evaluate the quality of the prepared material, the booklets were provided to four specialists in sleep disorders. They were asked to comment on the accuracy and comprehensibility of the content (information adequacy). Then, according to the specialists’ comments, the required changes were made. Subsequently, in a focus-group, the final content was provided to the specialists to check for the desirability of statements in terms of clarity (use of simple and comprehensible words) and use of common language (avoidance of technical and specialized terms).

Finally, considering the specialists’ comments, as it was possible that some patients would not undergo for the sleep test, the “healthy sleep habits” section was added to the sleep apnea booklet. The reason was to familiarize patients, even those who would not go for the test, with behaviors that can lead to a healthy sleep or cause insomnia. Moreover, considering the interviews with patients in the first phase and their questions on the treatment process, it was decided to include a brief description of therapeutic alternatives in the existing content of the booklet. As specialists insisted, these descriptions should simultaneously remove ambiguity about the therapeutic alternatives and not highlighted the complexity of the medical procedure that might impede patients’ adherence to prescribed sleep study. Moreover, as suggested by the specialists, the procedures involved in the sleep test described in the booklet were rewrite based on common activities and services in clinics. As an instance, the time it takes for the interpretation results varied across clinics. Besides, a brief explanation was added to the back cover of the booklet about the issue just raised.

To receive comments from the target patient population, an interview was held with a sample of that population to check the difficulty level of phrases and statements and lower the probability of ambiguity and misleading interpretations of the phrases and meanings. In case any problem arose, the changes were made to the content.

According to the content analysis of patients’ comments, some explanations were added to some terms (e.g. nocturnal enuresis in the sleep apnea symptoms section) to remove ambiguity. In the disease definition section, some specialized terms were omitted or were replaced by equivalents that were better comprehensible. As some participants were elderly, larger size fonts were used.

**Fig 2:**
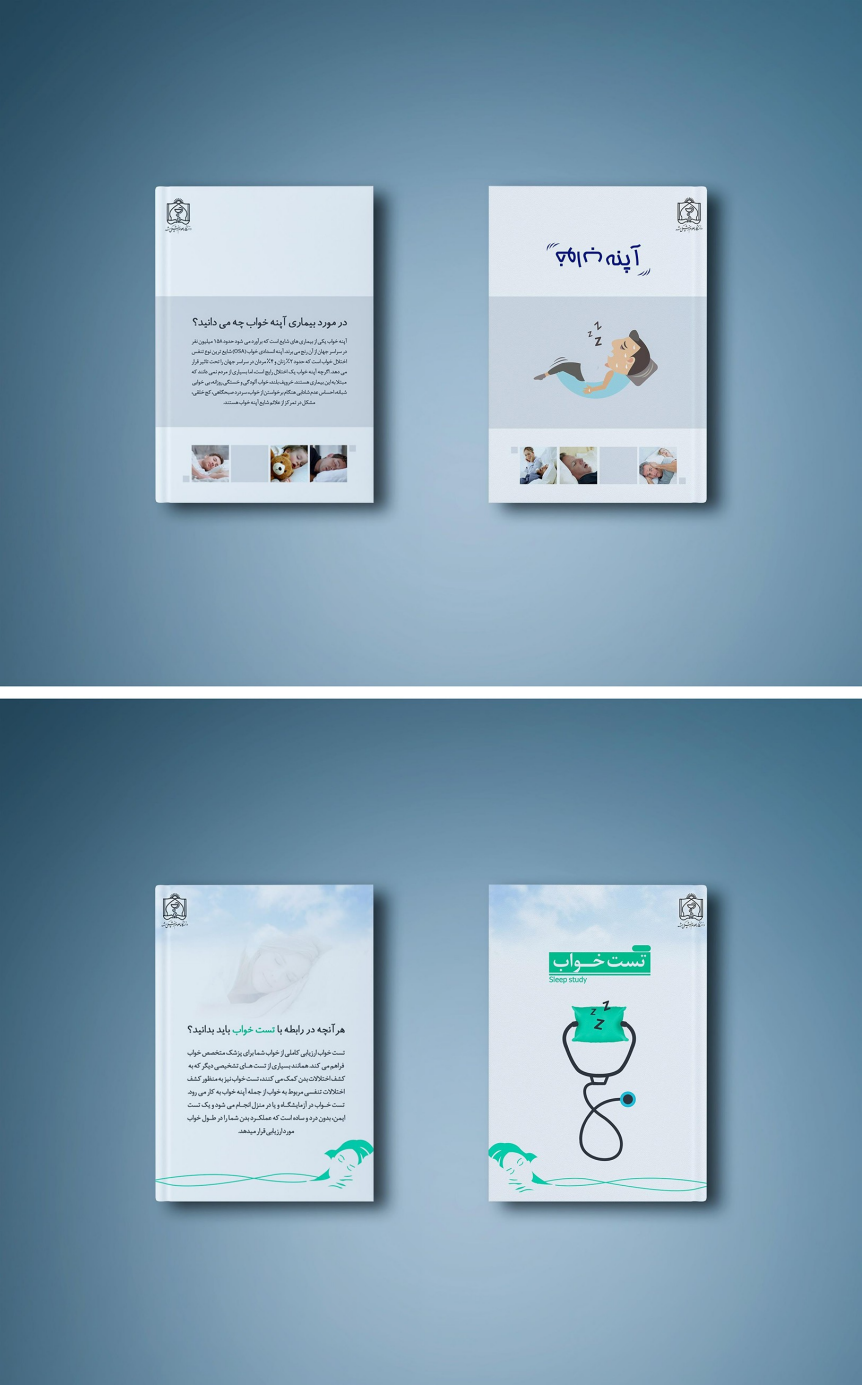
a)"Sleep Apnea" booklet b)"Sleep Study" booklet.

Accordingly, the sleep apnea booklet was developed in A5 paper size in 10 pages (Fig 2A) and the content covered:

- Definition of disease (overview and fact)
- OSA symptoms
- OSA Risk factors
- OSA consequences
- Brief explanation of sleep apnea diagnosis and treatment
- Healthy sleep habits

The sleep test booklet was developed in A5 paper size and 7 pages (Fig 2B). Its content covered the following:

- Benefit of OSA evaluation
- Types of sleep tests and an indication of each (implication and limitation) (In-lab sleep study and home sleep apnea testing)
- Describing the device, sensors, and environment
- How to prepare for the test
- Testing process
- How to analyze and interpret the results

### 2.7. Outcomes

All participants were contacted at least two months later to ask whether they completed an assessment for OSA. Patients were considered adherent for sleep testing if they underwent sleep testing. The adherence rate to prescribed sleep test was considered as the primary outcome.

Also, telephone interviews were conducted with patients in the intervention group who had not undergone the sleep test.

### 2.8. Sample size

In the present research, 50 participants were assigned to the control and 50 to the intervention group. Considering the differences between and among medical centers, physicians’ behaviors in treatment, to prevent any biased results, an equal number of patients from each center were selected for the control and intervention groups. In this way, for each center, 26 patients were included in each control and intervention group (n = 13 for each). Consequently, as four centers were investigated, a total number of 104 participants were included in the control and intervention groups.

### 2.9. Blinding

The data manager (who generated the randomization sequence, prepared envelopes, and maintained a list of enrolled participants), coordinators (who randomly assigned participants to control and intervention groups), and outcome assessor (who contacted and interviewed patients via telephone) were not blinded to participant assignment. Physicians and the data analyzer were completely blinded to the control and intervention group specification.

### 2.10. Statistical analysis

The normal quantitative variables (based on Kolmogorov-Smirnov normality test) were described using mean and standard deviation. The remaining data were described using median and interquartile range. To test the mean difference of quantitative variables between the control and intervention groups, if the normality condition was met, an independent-sample T-test was used. Otherwise, Mann-Whitney U-test was used to compare the data. To test the homogeneity of the qualitative variables in the two groups, Chi-squared test and Fisher’s exact test were used at the p-value of 0.05. Moreover, to compare the adherence rate to sleep test in both groups, Chi-squared test was used. To determine the predictors of the sleep test administration, logistic regression analysis was run. Therefore, first, univariate logistic regression analysis was run and variables with a p-value lower than 0.2 were detected. Then all these variables went for multivariate regression analysis in which the p-value was set at 0.05.

## 3. Results

**Fig 3:**
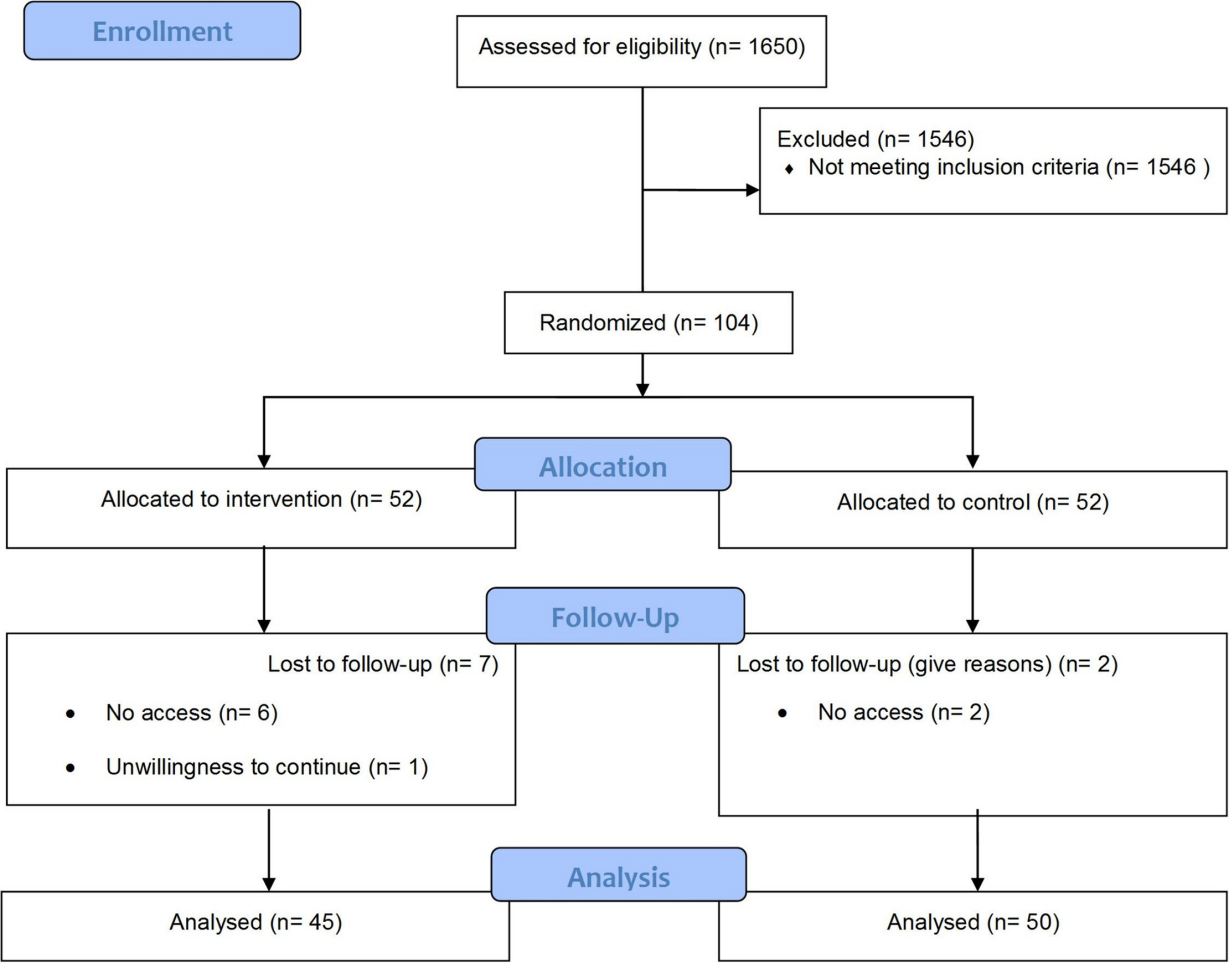
Consort flow diagram.

A total number of 1,650 individuals were screened for the RCT between January 2018 and August 2018. Among these, 1,546 did not meet the inclusion criteria. Finally, 104 patients were included in the study. CONSORT flow chart of patient recruitment is shown in Fig 3.

### 3.1. Differences between intervention and control

The patient participants in this research were examined in terms of demographic features, ESS scores, STOP-BANG scores, and medical history at baseline. As shown in Table 2, the research groups did not differ significantly in terms of the target features. It is noteworthy that the p-value of logistic regression in this table is concerned with the analysis of outcome.

**Table 2:** Demographic characteristic of participants in research groups.

**Variable**	**Intervention group (n = 52) (Mean ± SD)**	**Control group (n = 52) (Mean ± SD)**	**P-value**	**P-value (Logistic regression)**
Age (years)	47.5 ± 12.9	46.2 ± 11.9	.578 ^a^	0.03 ^e^
BMI (kg/m2)	30.5 ± 4.44525	30.2 ± 4.8	.761 ^a^	0.063 ^e^
ESS total score	9.01 ± 4.9	9.09 ± 4.8	.936 ^a^	0.470
STOP-BANG	4.6 ± 1.3	4.5 ± 1.2	.646 ^a^	0.733
**Variable**	**Number (%)**	**Number (%)**	**P-value**	**P-value (Logistic regression)**
Sex				
*Male*	35 (67.3)	33 (63.5)	0.680 ^b^	0.695
*Female*	17 (32.7)	19 (36.5)		
Marital Statues				
*Single/divorce/widow*	8 (15.4)	10 (19.2)	0.604 ^b^	0.802
*Married*	44 (84.6)	42 (80.8)		
Level of Education				
*Primary school*	14 (26.9)	18 (34.6)		
*High school/ Diploma*	19 (36.5)	16 (30.8)	0.676 ^b^	0.829
*Academic*	19 (36.5)	18 (34.6)		
Employment Statues				
*Governmental*	6 (11.5)	7 (13.5)		
*Non-Governmental*	27 (52)	29 (55.8)	0.676 ^b^	0.753
*Retired*	8 (15.4)	4 (7.7)		
*Housewife*	11 (21.1)	12 (23)		
Blood pressure				
*Yes*	19 (36.5)	15 (26.8)	0.403 ^b^	0.889
*No*	33 (63.5)	37 (71.2)		
Diabetes				
*Yes*	9 (52.9)	8 (15.4)	0.791 ^b^	0.144
*No*	43 (82.7)	44 (84.6)		
Stroke				
*Yes*	1 (1.9)	3 (5.8)	0.618 ^c^	0.843
*No*	51 (98.1)	49 (94.2)		
Coronary heart disease				
*Yes*	4(7.7)	3 (5.8	1.0 ^c^	0.159 ^e^
*No*	48 (92.3)	49 (94.2)		
Vascular Failure				
*Yes*	1(1.9	1(1.9)	1.0 ^c^	0.999
*No*	51(98.1)	51(98.1)		
Heart Attack				
*Yes*	0	0	- ^d^	-
*No*	52(100)	52(100)		
Angiography				
*Yes*	4 (7.7	7(13.5)	0.339 ^b^	0.592
*No*	48 (92.3)	45 (86.5)		
Fibrillation				
*Yes*	1 (1.9)	1 (1.9)	1.0 ^c^	0.344
*No*	51 (98.1)	51(98.1)		
Mood Disorders				
*Yes*	20(38.5)	22 (42.3)	0.689 ^b^	0.362
*No*	32 (61.5)	30 (57.7)		
Anxiety				
*Yes*	13(25)	15(28.8)	0.658 ^b^	0.200 ^e^
*No*	39(75)	37(71.2)		
history of similar illnesses in the family				
*Yes*	26(50)	22(42.3)	0.431 ^b^	0.105 ^e^
*No*	26 (50)	30(57.7)		
Car accident due to sleepiness				
*Yes*	6 (11.5)	1 (1.9)	0.112 ^c^	0.615
*No*	46 (88.5)	51 (98.1)		
Nocturnal enuresis				
*Yes*	11(21.2)	10 (19.2)	0.807 ^b^	0.163 ^e^
*No*	41 (78.8)	42 (80.8)		
Morning Headache				
*Yes*	15 (28.8)	18 (34.6)	0.527 ^b^	0.364
*No*	37 (71.2)	34 (65.4)		
Difficulty in Focus				
*Yes*	24 (46.2)	21 (40.4)	0.553 ^b^	0.488
*No*	28 (53.8)	31 (59.6)		
Memory loss				
*Yes*	13 (59.1%)	9 (40.9%)	0.337 ^b^	0.782
*No*	39 (47.6%)	43 (52.4%)		
Decrease in libido				
*Yes*	21 (40.4)	21 (40.4)	1.0 ^b^	0.423
*No*	31 (59.6)	31 (59.6)		
petulance				
*Yes*	23 (44.2)	23 (44.2)	1.0 ^b^	0.232
*No*	29 (55.8)	29 (55.8)		
Snoring				
*Yes*	47 (90.4)	41 (78.8)	0.103 ^b^	0.458
*No*	5 (9.6)	11 (21.2)		
ESS				
< = 10	31 (47.7%)	34 (52.3%)	0.843 ^b^	0.102 ^e^
> 10	21 (53.8%)	18 (46.2%)		
STOP-BANG				
< 3	31 (59.6)	34 (65.5)	0.543 ^b^	0.602
> = 3	21 (40.4)	18 (34.6)		

^a^Results of the t test

^b^Results of the χ2 test

^c^Results of the Fisher exact test

^d^No statistics are computed because HeartAttack is a constant

^e^ P < 0.2

**Fig 4:**
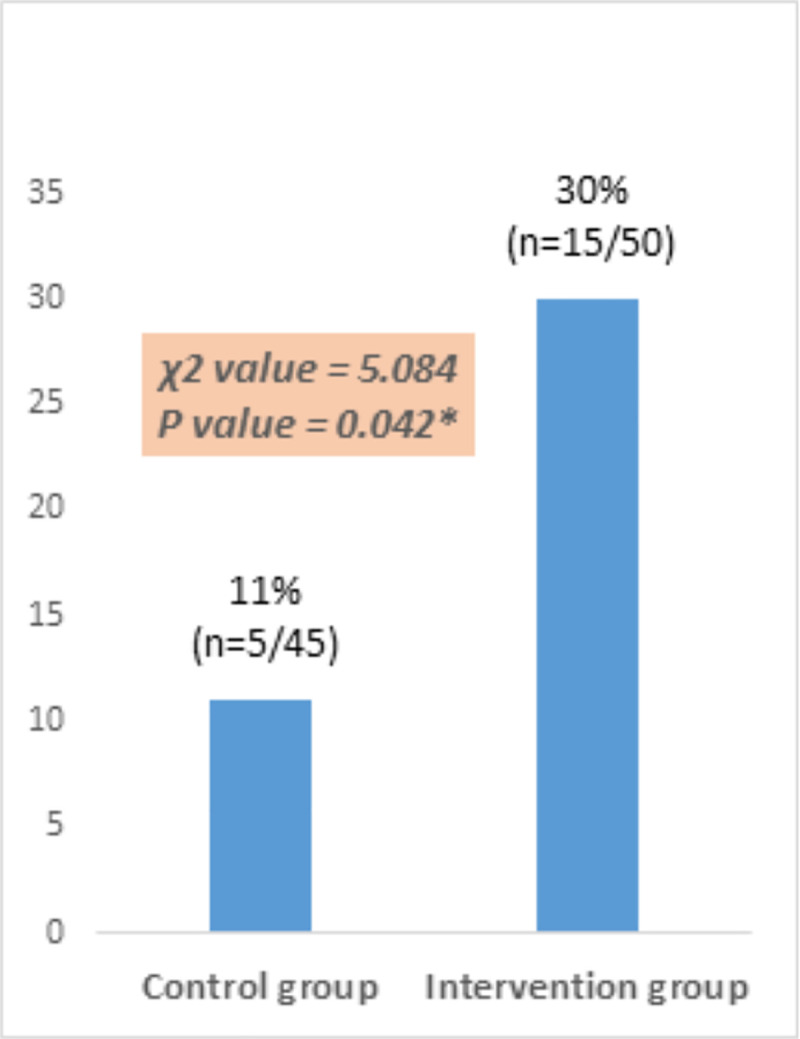
Comparison of control and intervention groups in terms of the adherence rate.

χ^2^ analysis was used to examine whether individuals randomized to the intervention arm were more likely to attend a sleep assessment for OSA risk than those in the control arm. The results of the intention to treat analysis indicate that respondents who received the educational intervention were significantly more adherent to attend a sleep assessment for their OSA risk (30%; n = 15/50) than those who did not receive the intervention (11.1%; n = 5/45) (χ2 = 5.084, P <0.05) (Fig 4).

To identify the predictors of outcome, logistic regression was run. In univariate logistic regression, a significant relationship (P<0.2) was observed between age, BMI, diabetes, Coronary, anxiety, Family History, Enuresis, ESS as well as being in control/intervention groups (P-value: 0.03, 0.063, 0.114, 0.159, 0.201, 0.105, 0.163, 0.102, 0.029) and adherence to physician-recommended evaluation. The P-values obtained from the univariate regression analysis are presented in Table 1.

Multiple logistic regression analysis was performed to examine the effect of these variables on adherence to physician-prescribed test. As shown in Table 3, Age, diabetes, and being in the control/intervention group had a significant relationship (P<0.05) with adherence to physician-prescribed test. Patients with higher age, lower BMI, and provided with educational materials were more likely to attend a sleep assessment for OSA risk.

**Table 3:** Multivariate logistic regression analysis results of the correlation between potential influential factors and sleep test administration.

Variable	Confidence interval	P value
**Age**	1.01–1.12	0.011*
**diabetes**	1.02–87.04	0.047*
**Receiving educational intervention**	1.19–13.8	0.025*
**Family history**	0.695–7.467	0.174
**Anxiety**	0.138–1.804	0.289
**Nocturnal enuresis**	0.257–5.831	0.800
**Coronary disease**	0.092–5.984	0.780
**ESS**	0.139–1.422	0.172
**BMI**	0.781–1.005	0.060

*P < 0.05

### 3.2. Reasons for patients’ not adhering to physician’s prescription of sleep test

To find out why patients do not adhere to physician’s prescription of sleep test, an interview was held with patients in the intervention group (those who had not undergone the sleep test). Twenty-two participants of the total number of 35 were willing to be interviewed. The reasons why they had not adhered to physician’s prescription of sleep test are summarized in Table 4.

**Table 4:** Reasons of patients in intervention group for not adhering to physician’s prescription of sleep test.

Reason	Frequency
Time limitations due to job-related commitments	15
Condition improvement	11
High cost of diagnostic test	5
Referral to another physician	4
Travel	4
Geographic distance to the facility	3
Dissatisfaction with hospital services and personnel (to make an appointment)	3
Inadequate knowledge of disease symptoms	2
Not taking the disease and its consequences seriously	2
Time-consuming process of test administration	2
Lack of insurance coverage	1
Forgetfulness	1
Lack of family satisfaction	1
Discomfort with an unfamiliar environment	1
High cost of treatment device	1
Not accepting the disease	1
Not prioritizing the sleep test over other medical condition	1
Not prioritizing the sleep test over other medical fees	1

## 4. Discussion

In the present research, attempts were made to explore the effect of an educational intervention on patients’ adherence to physician’s prescription of sleep test. The present results revealed that those who received the educational intervention had significantly more adherence to sleep study testing than those who did not receive the intervention. This would point to the fact that the intervention designed to remove barriers to sleep test administration with the aim of raising the level of knowledge and removing patients’ concerns and ambiguities (psychological factors) managed to be effective. In similar studies that also used educational interventions to meet the needs of the target population and were personalized according to the culture and language of the target population, the result was effective on behavior change [17, 20].

The logistic regression results showed that the variables age, affliction with diabetes, and reception of intervention were the predictors of adherence to physician’s prescription of sleep test. As the regression analysis showed, higher age could lead to better adherence [16]. It seems that those of a higher age show more sensitivity to their health state and, therefore, adhere to physician’s prescriptions more.

Another influential factor in adherence to physician’s prescription of sleep test was affliction with diabetes. Those with a history of diabetes stood more chances of test administration than the control. It seems that those with the experience of chronic disease accepted the disease and the current condition more than others. Those without the experience of a chronic disease had concerns that they would be diagnosed with a chronic disease through the sleep test [13].

Moreover, it can be embarrassing to some others. Thus, they show no interest in administering the test. Raising the awareness of these individuals and the rest of society of the serious consequences of the disease can cut down on delayed test administration and lead to timely treatment. Contrary to the present research, findings from other investigations showed no effect of diabetes, obesity, or hypertension on sleep test administration [20]. Yet, improvement in OSA can improve these conditions too [21–23].

One key variable affecting the adherence rate was the reception of educational intervention. Patients who received the booklet stood more chances of administering the sleep test than others. In the light of the related literature, the majority of patients can recall less than 35% of information orally communicated by healthcare specialists. Furthermore, the patients might not understand all the content and might be too shy to ask questions. Thus, textual content can be used to complement and enhance orally communicated content [24]. The present findings which also took advantage of textual material to educate patients confirm this claim. Moreover, educational interventions raise awareness of OSA especially of the risk factors and symptoms of the disease. That would mean those with symptoms of the disease are more likely to ask relevant questions from the healthcare providers than others. Yet, the overall assumption is that the general awareness of OSA in the recent three decades has been constantly on the rise. However, a body of research has shown that awareness and knowledge of OSA in public are still at a low level. Thus, many cases of OSA are left undiagnosed [25, 26].

The analysis of reasons for patients’ not adhering to physician’s prescription in the intervention group showed time limitation as one of the most recurrent reasons, which was basically due to work loads. Moreover, some patients mentioned the long and time-consuming process of test administration as a barrier to having the test. It seems that inadequate knowledge has made some patients neglectful of the disease. For different reasons such as work load and overlaps with other tasks, they refrained from having the polysomnography [11, 12, 26].

Improvement of patient conditions was another recurrent reason mentioned for not adhering to physician’s prescription. Yet, there was no evidence documented for this and it may only have psychological roots and self-doubt. Another reason mentioned by the participants was the high cost of diagnosis and treatment. Some patients though were willing to have the test did not do so as the cost was not covered by the insurance company. It should be noted that the costs of sleep test and treatment procedures are not covered by insurance in Iran. In addition, low awareness at the society level has also paralyzed social supportive service providers such as charities and consultation offices. They are basically unaware of such diseases and thus do not prioritize contributions to sleep tests. Moreover, for patients who were afflicted with other diseases, the priority would go for other diseases than the sleep test. This could, in turn, originate from patients’ lacking awareness [26].

Another reason why several patients did not undergo the sleep test was referring to another physician and the second physician’s lack of adequate insistence on the necessity of the test. Trying another physician to confirm or reject the prior physician’s diagnosis can be a sign of distrust in the physician or generally distrust in the medical system. This point has been also raised in the related literature as a barrier to sleep test administration [14].

Among the reasons why some patients did not undergo the sleep test was their absence at their residence due to frequent trips. As the present research was conducted in summer, some patients who were visited during this time and were then contacted, did not undergo the sleep test for traveling reasons. The follow-up duration (two months after visiting the physician) was short in this research and some patients were likely to undergo the sleep test at a later time. Reminding patients, emphasizing on the necessity of the test and following up their condition at regular intervals might tackle this problem. This solution can also help patients who forget all about the test.

Geographical distance from the service providing center was mentioned as another reason for not adhering to the test. Due to costly polysomnography equipment and the need for an appropriate place for test administration, this test was not available everywhere. Thus, the geographical distance from the test administration center could be far [15]. Other related literature also raised this issue as a barrier to sleep test administration [26, 27].

Patients’ dissatisfaction with service provision was another reason for not going for the sleep test. As some centers were located within hospitals, the relevant staff might be overloaded with work and, thus, not much responsive to patients’ enquiries and expectations. This point was also mentioned in the related literature as a barrier to sleep test administration [12, 26].

Several patients refused to go for a sleep test because their family disagreed with this action. Part of the Islamic beliefs is to help those in need of help especially those with a disability or so. When Muslims get sick, they expect to be supported by close family members or relatives. A body of research has also proved the key role of social support in adhering to medications in different cultures [28, 29]. Therefore, it makes sense that patients lacking family and social support show less tendency to go for the sleep test [12]. Besides, certain moral, religious and cultural frameworks have shown to significantly influence adherence to medication [30]. As an instance, in Islam, health is values and any attempt to threaten health is condemned. Thus, if one does not adhere to medication and accordingly threatens one’s health, s/he is reprimanded in the culture. Patients are truly expected to try their best to gain health. In Iran, where the majority of the population is Muslim, people are expected to adhere more to physician’s advice than any other religion or country. Yet, this issue was not explored in depth in the present study and no decisive conclusion can be made accordingly for how spirits, religion and cultural values are related and how they affect the adherence rate to sleep test.

According to univariate logistic regression analysis, gender had no effect on the rate of adhering to the sleep test. Though for some women adherence can be lower due to it’s the dependence on family consent and financial/emotional support or some socio-cultural constraints, the quantitative analyses in this study did not confirm this.

There is a body of research in different disciplines that partially report a lower adherence to medication among women than men [31, 32]. Some others proved otherwise [33, 34]. In communities, where women stand lower chances than men, there is a need for the design of purposive interventions that take into account cross-gender differences. Presenting gender-specific content for each gender might increase the rate of adherence to medication.

The unawareness of family members and not taking the issue serious can be among the reasons for this lacking cooperation. Besides educating patients, educating influential individuals can affect patients’ decision; especially the spouse or children can affect the decision.

Though in the present research, the adherence rate of patients was higher in the intervention group than the control, yet a significant number of patients did not undergo the sleep test. It seems that making more complicated interventions such as personalized interventions based on each patient’s behavior can help with this regard. Moreover, many patients, despite suffering from severe respiratory problems during sleep, showed no respiratory problem while awake. Using videos and interviews with people with similar experiences would help with accepting this issue by patients.

### 4.1. Limitations

1. In the present research, the only test prescribed was polysomnography. For some patients, under certain circumstances, home-based domestic diagnostic tests can be used. Therefore, certain barriers to sleep test administration can be removed and the non-adherence rate of patients can be reduced.
2. The intervention effect was investigated in the short run. A two-month interval (since visiting the physician to the sleep test administration) was short and can have overestimated the non-adherence results among patients. Thus, a longer-term interval is required to investigate the adherence or non-adherence rate of patients.
3. Another limitation of the present research was the use of intention to treat analysis. It was assumed that patients in the intervention group received the educational booklet and perused it, while in fact that might not be true. Yet, the use of intention to treat analysis due to commonalities with routine social conditions can raise the generalizability of results.

## 5. Conclusion

The intervention applied in the present research with the aim of meeting the needs of the target population managed to improve the adherence rate of patients to sleep test. However, the adherence rate of patients was still far from satisfactory and requires more complicated interventions. Implementing educational and psychological interventions can help to raise patients’ awareness and remove their ambiguities and can, thus, increase the adherence rate. Moreover, attempts to lower costs through more insurance coverage, subsidies and promotion of the quality of services provided to patients can play a key role in increasing their willingness to go for the test.
